# The effect of local species composition on the distribution of an avian invader

**DOI:** 10.1038/s41598-019-52256-9

**Published:** 2019-11-01

**Authors:** Tali Magory Cohen, Roi Dor

**Affiliations:** School of Zoology, Faculty of Life Sciences, Tel Aviv University, Tel Aviv, 69978 Israel

**Keywords:** Ecological modelling, Invasive species

## Abstract

Estimating the potential distribution of invasive species has been primarily achieved by employing species distribution models (SDM). Recently introduced joint species distribution models (JSDM) that include species interactions are expected to improve model output. Here we compare the predictive ability of SDM and JSDM by modelling the distribution of one of the most prolific avian invaders in the world, the common myna (*Acridotheres tristis*), in a recent introduction in Israel. Our results indicate that including information on the local species composition did not improve model accuracy, possibly because of the unique characteristics of this species that include broad environmental tolerance and behavior flexibility. However, the JSDM provided insights into co-occurrence patterns of common mynas and their local heterospecifics, suggesting that at this time point, there is no evidence of species exclusion by common mynas. Our findings suggest that the invasion potential of common mynas depends greatly on urbanization and less so on the local species composition and reflect the major role of anthropogenic impact in increasing the distribution of avian invaders.

## Introduction

The growing concern with the impact of invasive species on the natural environment has recruited many methods to provide an insight into what habitats would potentially suite the invaders, including employing species distribution models (hereafter, SDMs)^1,2^. A large body of evidence has already been generated to this end, from plants^1,3–5^, to invertebrates^6–8^, amphibians^9–11^, reptiles^12–14^, mammals^15,16^ and birds^17–19^. The majority of this work makes use of a-biotic predictor variables in order to characterize the environmental conditions of where the species is found^20^. The output of these studies is eminent in providing a practical, useful data which can benefit management practices on varying geographic scales^21^.

However, the exclusion of biotic predictors from these models has been widely criticized^20–22^. The term ‘biotic interactors’ may relate to relevant competitors, predators, consumers, parasites, pollinators or hosts^20^, and an increasing number of empirical evidence suggests that their effect is not limited to a local extent^20,23–26^. The main hindrance in including biotic interactors remains the accuracy of the data, which can often be too coarse or simply missing^21^. When such data is available, one may choose how to incorporate it into the model (see Anderson^20^ useful guidelines). Among others, relevant species can be modelled as variable predictors as either present or absent in a certain cell, thus relating to them only in a unidirectional manner^25^. Conversely, Joint Species Distribution Models (JSDM) consider multiple species simultaneously taking into account the multidirectional nature of species co-occurrence patterns, which can be driven by inter-species interactions^22^. These models estimate a matrix of pair-wise correlations between species, allowing inference of residual co-occurrence among species^27^. By integrating species co-occurrences in the model or during post-processing, model accuracy is expected to increase, and model predictions can be improved^20^.

In order to study the effect of including biotic interactions on assessing the distribution of an invasive species in its introduced range, we explored the well-documented introduction of the common myna (*Acridotheres tristis*), a broad-ranging avian invader, into Israel. This invasion is fairly recent (since 1997; Holzapfel *et al*.^28^), and occurred over a very heterogeneous landscape which includes four phytogeographic areas^29^, four Köppen climate classifications^30^, altitude ranging from (−430) to 2,814 meters and diverse communities of animal and plant species^31,32^. The richness of environmental features that exist in a small area allows for studying the invasion process over different ecosystems and represents many of the environmental conditions previously found to be important in determining the species global distribution^33^. Despite the relatively short time that has passed since the introduction, common mynas have reached all ends of the country and beyond (invading neighbouring Lebanon and Jordan; Ramadan-jaradi^34^, Khoury and Alshamlih^35^), and we therefore treat them as being at equilibrium at the study domain. Additionally, high resolution environmental data pertaining to both a-biotic and biotic variables (e.g., landscape features, native species composition) enables high quality data that can be complemented by the well-documented invasion of the common mynas. The accuracy of SDMs depends greatly on the spatial resolution and extent of the data^36^. Although coarse resolution studies have been found to be informative^37^, model performance usually increases with resolution and extent^36^.

Our goal was to evaluate the role of species co-occurrences in predicting species distributions of invasive species by comparing high-resolution SDMs and JSDMs, using the case study of the common myna invasion into Israel. By doing so, we describe the range expansion of common mynas in Israel and the factors that influence it, subsequently identifying potential habitats for common myna presence in Israel. We expect that combining local species composition data will improve model accuracy and benefit model predictions. The results of this study will aid in establishing the significance of biotic interactors in estimating the potential distribution of invasive species and inform management practices. This study will also illustrate the factors that shape the range of this important avian invader.

## Results

**Figure 1:**
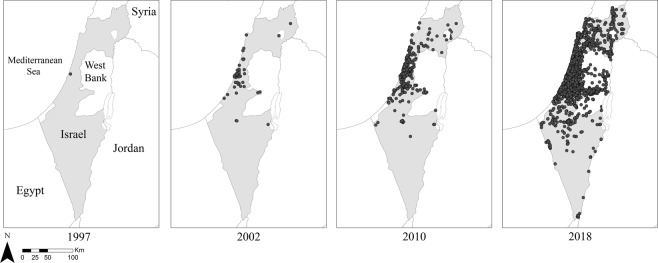
Occurrence records of common mynas in Israel used in this study during four different periods: the year of invasion, 1997; until 2002; until 2010 and until 2018. Presence records are marked with black circles.

In this study, we collected occurrence data on one focal species, the common myna (n = 762, after spatial filtering), and 12 local species that are its direct or indirect competitors (Supplementary Table S2). Over the course of ca. 21 years since the introduction event, common myna distribution has increased to include the majority of the country (Fig. 1). The spread of the mynas was asymmetrical and occurred initially along the coastline and northwards. However, while sporadic and slower, the species spread into the south of the country shortly after. In 2018, the distribution of the common myna in Israel is considered to have reached all of the country’s borders with its neighbouring countries, and sightings of common mynas in Lebanon and Jordan have been reported, possibly due to range expansion from Israel.

The results from the MaxEnt model indicate that the environmental variable that contributed the most to the model was Impervious Surfaces, an anthropogenic factor that quantifies constructed impervious surface areas [km^2^] (79.1%). The remaining explanatory variables, mostly a-biotic predictors, explained a significantly lower percentage of the model variability (Table 1). Whereas the logistic prediction probability of presence increased logarithmically with the anthropogenic predictors (IMP, HD), most of the a-biotic variables climaxed at a single optimal value (bio11, bio17, Supplementary Fig. S1). The range of mean temperatures of the coldest quarter (bio11) that correlated with a high probability of presence (>0.5) was relatively wide (12.5–15 °C) and suggests that mynas can persist in areas with a broad temperature range.

**Table 1:** Variable contribution measured in percentages as generated by the MaxEnt model.

Variable	Percent contribution
Impervious surfaces	79.1
Mean Temperature of Coldest Quarter (bio11)	9.7
Temperature Seasonality (standard deviation *100) (bio4)	4.5
Precipitation of Driest Quarter (bio17)	4.3
Human Density	2.4

**Figure 2:**
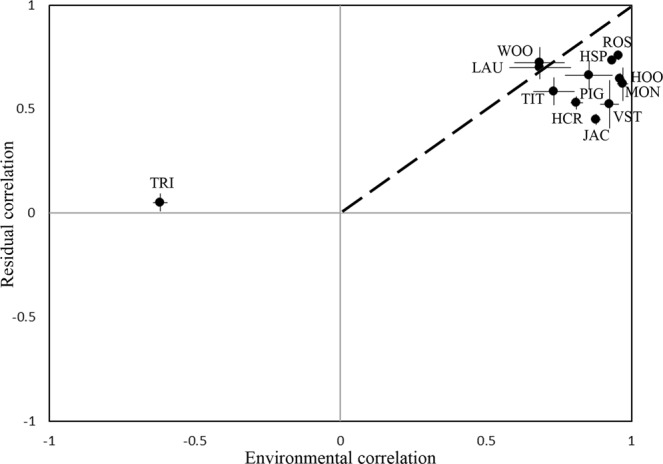
Modelled residual correlation and environmental correlation between common mynas and local species. Error bars represent standard deviation. Species abbreviations are detailed in Supplementary Table S2. Strong positive residual correlation suggests biological interactions between species, whereas that environmental correlation could indicate similar habitat preference. Dashed line represents a 1:1 trendline.

Unlike the SDM, the JSDM allows computing individual regression coefficients of the probit model of each predictor. However, interpretation of these coefficients is difficult because the increase in probability depends both on the values of the remaining predictors and the intercept (Table S3). Nevertheless, it enables separating the effect of the environmental predictors from those of the species co-occurrences, which indicated that most species responded similarly to environmental conditions and had positive residual correlations in co-occurrence with the mynas, except for Tristram’s starling (*Onychognathus tristramii*), which occupied a different environment and had near-zero residual correlation (Fig. 2). Negative estimates of environmental correlation indicate that common mynas and Tristram’s starlings rarely co-occur because they occupy different habitats rather than because of species interactions. Generally, environmental correlations were higher than residual correlations.

Comparison of the two models (SMD and JSDM) was achieved by calculating a threshold-independent discrimination measure (AUC) and by applying the Maximum Sensitivity plus Specificity threshold to calculate model sensitivity. AUC scores were high for both models and nearly identical (0.897, JSDM; 0.906 ± 0.04, MaxEnt). Conversely, sensitivity values varied slightly more between the models. Although sensitivity scores differed between the five cell sizes, they peaked at 84.78% in the MaxEnt model and at 80.58% in the JSDM (Table 2).

**Table 2:** Model performance as measured by AUC (Area Under the Curve) and sensitivity. Sensitivity was calculated by applying the Maximum Training Sensitivity Plus Specificity Threshold for five cell sizes. The Maximum Training Sensitivity Plus Specificity Threshold is the average value calculated over 100 iterations.

	JSDM					MaxEnt				
Cell Size	0.0004	0.01	0.02	0.03	0.05	0.0004*	0.01	0.02	0.03	0.05
AUC	0.897					0.906 ± 0.04^^^				
Maximum Training Sensitivity Plus Specificity Threshold	0.196					0.264				
Sensitivity	80.45	80.18	80.58	80.58	80.84	84.78	84.78	79.66	76.12	75.33

*Original cell size used by the algorithm.

^^^Standard deviation.

**Figure 3:**
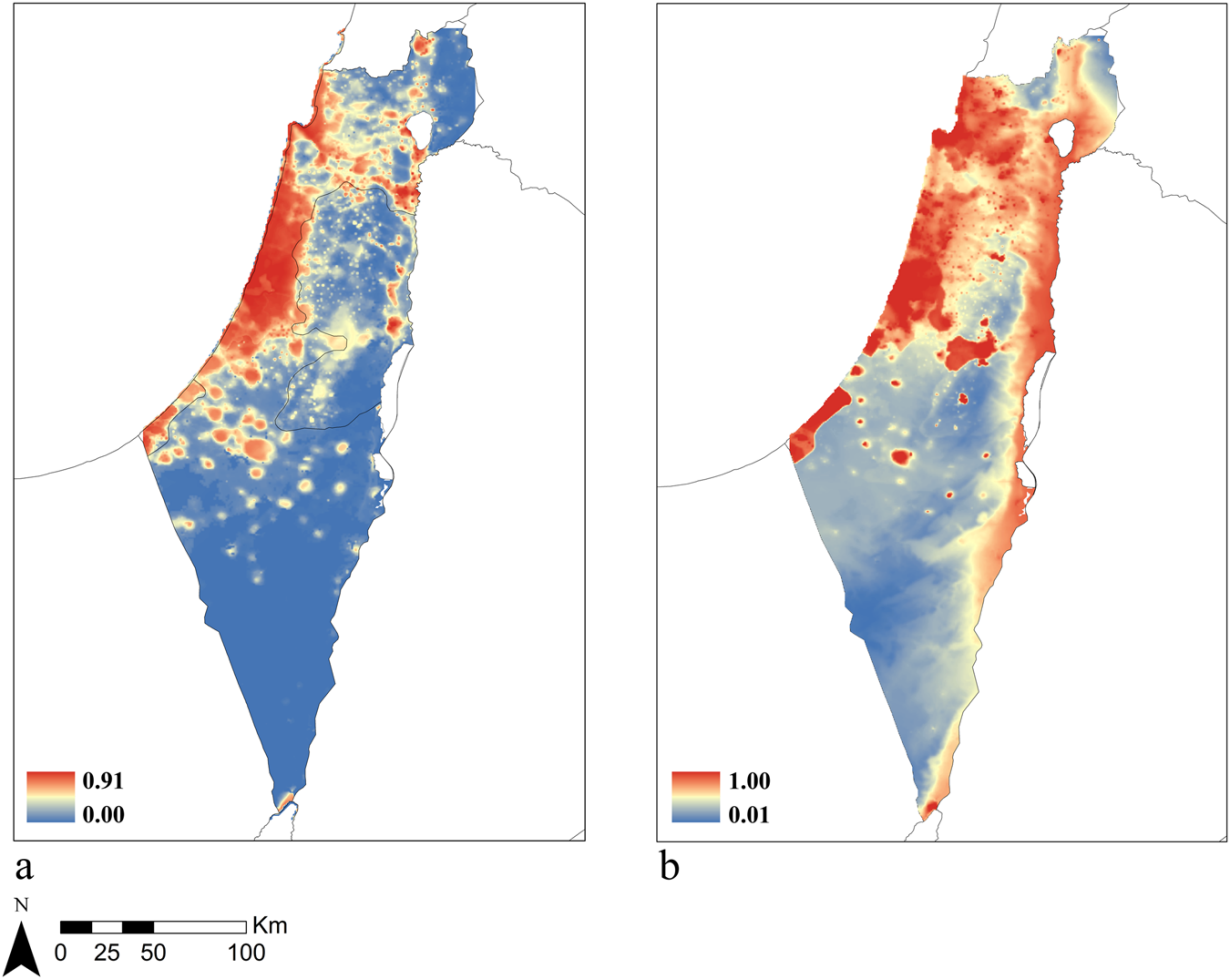
A continuous map of average probabilities for suitable areas for current and potential common myna presence based on the MaxEnt model (**a**) and the JSDM (**b**). Legend contains colour code representation of probabilities.

Continuous maps of suitable habitats for each model showed similar, but not identical, results (Fig. 3). Both models showed that major parts of the country are suitable for common myna presence, apart from high-altitude, unpopulated areas and unpopulated parts of the Negev desert. However, while model accuracy indices did not favour the JSDM (see Table 2), the continuous map generated by extrapolating its model output included larger areas throughout the country, chiefly in the Jordan Rift Valley and in the north.

## Discussion

This study aimed to describe the past, current and potential distribution of the Common Myna in Israel, a new introduction site for this prolific avian invader. By using high-resolution data and accurate species records achieved by extensive sampling, we were able to compare the accuracy of two models: MaxEnt, which utilizes only environmental predictor variables, and a JSDM, as described by Pollock *et al*.^22^, which also incorporates species co-occurrences. The results of these models also allowed us to illustrate the potential suitable habitats in the few areas that have yet to be invaded by mynas in Israel and offer an insight into the facilitating factors that mediate this spread.

The results of the MaxEnt model and the JSDM did not differ significantly. Both models performed well and described similar potential suitable habitats, suggesting that species co-occurrences don’t play an important role in determining the suitability of the new environment to common myna invasion in Israel. Despite occurring through diverse habitats across the country, this finding was consistent, highlighting the significance of the environmental features over species composition. We postulate that the minor contribution of species co-occurrence to explaining the current distribution of common mynas in Israel is a result of the species’ behaviour flexibility and broad environmental tolerance^33,38–40^, which allow it to occupy various environments regardless of the local species composition. This result is also echoed in the broad global range this species occupies, that spans multiple ecosystems and vastly different communities^33^. Since the level of urbanization (as reflected in the area of impervious surfaces) had the largest contribution to the model, it is likely that common mynas favour urbanized areas, where species are expected to display higher levels of neophilia and innovation^41^. However, our study did not consider species abundance nor was it intended to estimate the effect of common mynas on local species, which require a different study design. Additionally, the inclusiveness of the continuous map resulting from the JSDM, suggests an existing additive effect of including local species which may offer further insight in future studies that include abundance data. Although the additional areas included in the JSDM predictions but not in the SDM’s map do not offer an improvement in model accuracy based on current records, we stress that they should be considered as potential ranges because invasive species management practices require maximum caution and an expectation for the worst-case scenario.

Species co-occurrence patterns detected by JSDM indicated that most species shared similar environmental preferences and had positive residual correlations in co-occurrence. This suggests that common mynas occur mutually with other species, although it does not necessitate actual interactions. In Israel, common mynas often forage in heterospecific groups along with house sparrows, domestic pigeons, laughing doves, Eurasian hoopoes, hooded crows, monk parakeets and more. Similarly, previous studies that the presence of common mynas at a feeding resource did not negatively affect the abundance of heterospecific individuals compared with other species with which they co-exist^42,43^. Moreover, house sparrows decrease their vigilance while foraging in proximity to common mynas, possibly because they benefit from mynas’ alarm calls (I. Berger, unpublished data). Conversely, common mynas have been shown to displace local birds at nesting cavities^44,45^, which may result in further displacement from the area. It is possible that because the invasion of common mynas into Israel is very recent (approximately 21 years ago), evidence for such exclusion has yet to be found and that further assessment is required in the future. An alternative reason for the observed pattern of positive co-occurrence is that, given computational limitations, the study design included the most relevant local species, i.e., potential competitors for nesting cavities or for similar foraging preferences. Our preliminary data suggests that, whereas adding less relevant species increases the variability of environmental co-occurrence, residual co-occurrence stays close to zero in most species. Conversely, without direct evidence to quantify interspecific interactions among the studied species, we cannot rule out that the residual correlation patterns observed here reflect unmeasured covariates.

The spread of the common myna in Israel has been rapid and geographically unbalanced, with minimal or no lag stage. While it is evident that the arid south of Israel was invaded last, temperature explained only a small part of the current distribution. Alternatively, the delay in the expansion of the common mynas into the south of Israel is because it is less populated. Equivalently, the anthropogenic factor (impervious surfaces, human density) had the largest contribution to explaining the current myna distribution, and was positively correlated with the probability of myna presence. This finding indicates that urbanization is an important mediating factor in the spread of this avian invader in Israel, complementing results obtained on a global scale^33^. Moreover, by eventually spreading across the Israeli heterogeneous landscape, including its southern desert, common mynas demonstrate their ability to invade areas where the environmental conditions are different than their natural range, possibly through the aid of urbanization. Traditionally, however, invasive species are presumed to succeed mainly in introduced areas that share similar environmental characteristics with their original niche^46^. Contradictorily, despite occupying mainly rural or semi-urban areas in their native range, common mynas have both become accustomed to the urban environment and learned to employ it to spread into environments that may otherwise be different than their native one.

These findings strongly indicate that the invasion potential of common mynas depends greatly on urbanization and less so on the local species composition, explaining in part their global invasion success. The velocity of the spread and recent reports of recordings from neighbouring countries (Jordan^35^ and Lebanon^34^) are indicative of the invasiveness of this species, raising concern for additional regional range expansion as well as a global one. Our results can inform management efforts aimed at controlling local spread by delineating regions at risk, as well as emphasizing the need to prevent additional introductions through the bird trade. We highlight the need that future research effort focus on assessing the ecological impact of common mynas in order to maintain species diversity in an environment that is constantly becoming more urbanized.

By comparing the traditional method of modelling the distribution of invasive species (SDM) with the emerging JSDM, we show that in the case of the common myna, a high-risk potent invader, including information on local species composition did not improve model accuracy, thus providing empirical evidence as to the effectiveness of traditional SDMs compared to JSDMs. However, these models are not identical in their formulation. While previous studies have successfully compared different models^47–53^, including JSDM^54^, these results require caution in interpretation. In addition, the use of pseudo-absences in JSDM has yet to be tested. Despite that, our study demonstrates that a change of paradigm should be considered for wide-spread invasive species that are more likely to be unaffected by the local species community. Our results can be used to optimize mitigation strategies and pre-emptive assessments of invasion potential of other non-local species.

## Methods

### Data collection and preparation

In order to obtain high resolution presence-only occurrence data, we initiated a citizen science project aimed at collecting common myna occurrence data in Israel in collaboration with The Israeli Centre for Yardbirds (ICYB). The project was founded in 2015 and has been ongoing since, through advertising in local media, education entities and social networks. It engaged professional and non-professional birders to report the location, date and time, quantity, habitat type and activity of common mynas. This allowed for high resolution record collection which would otherwise require exhaustive resources. To complement that, we utilized additional sources which included publicly available online databases, a governmental agency (Israel Nature and Parks Authority), museums, private organizations (ICYB, the Society of Protection of Nature in Israel - Israel Birding Portal, HaMaarag – Israel’s National Ecosystem Assessment Program), and previous studies (a full list of resources is available in Supplementary Table S1). In addition, we collated occurrence data of 12 local species (both native and other aliens) that were of potential biological importance to the presence of common mynas, such as direct/indirect competition for food resources, nesting cavities or proximity to humans (Supplementary Table S2). Because of computational limitations, the species selected included those that have been previously reported to occupy similar niches or displayed similar commensal behaviour^44,45,55,56^. These data were obtained from the ICYB, the INPA, Israel Birding Portal and HaMaarag. These local species occurrences were used as response variables only in the JSDM.

Data preparation included georeferencing of records according to the known locality (if the original coordinates were missing or erroneous) and removing duplicates. We employed spatial filtering in order to account for bias stemming from sampling efforts (see ‘Environmental modelling’), and a final data set of samples collected in the years 1997–2018 was generated for each species, ranging between 133–7,181 records per species (Supplementary Table S2). We also corrected for spatial autocorrelation by applying a filter of a minimum distance of 3 km to the occurrence records as shown by Magory Cohen *et al*.^33^. Because of the political nature of the region, sampling effort was lower in the West Bank territories (n_myna_ = 105) and the Gaza Strip (n_myna_ = 0) to which common mynas are also assumed to have spread. While the sampling effort was low, the unique landscape and environmental conditions of the West Bank area seemed important to the analysis. Therefore, we ran a subset of the data excluding the data points in the West Bank from occurrences and subsequent spatial analyses (n_myna_ = 652). Model outputs were highly similar, and so we present the results of the complete dataset.

### Environmental variables

We selected a set of environmental variables that included both biotic and a-biotic factors that may be of biological importance to common myna distribution in Israel following Magory Cohen *et al*.^33^. A-biotic environmental predictors included isothermality (mean diurnal range/temperature annual range) (* 100; bio3), temperature seasonality (standard deviation *100; bio4), mean temperature of warmest quarter (bio10), mean temperature of coldest quarter (bio11), precipitation of wettest month (bio13) and precipitation of driest month (bio14), precipitation of wettest quarter (bio16) and precipitation of driest quarter (bio17) (http://chelsa-climate.org/)^57^, as well as Human density (HD) (https://sedac.ciesin.columbia.edu/)^58^, impervious surfaces (IMP) (https://ngdc.noaa.gov/)^59^, percentage of vegetation (http://www.hamaarag.org.il/)^60^, altitude (ASTER 2, METI/NASA, 2011, http://due.esrin.esa.int/) and resources availability in the form of Actual Evapotranspiration (AET; the CGIAR-CSI Global Soil-Water Balance Database^61^, available at http://www.cgiar-csi.org).

We employed a unified spatial resolution of 0.0004 cell size (approximately 50 meters) by resampling (‘raster’ package; Hijmans^62^). The extent chosen for the study depended greatly on the availability of the data and was therefore limited to Israel. We corrected for sampling bias by filtering presence records according to the average foraging flight distance of each species (Supplementary Table S2). The purpose of spatial filtering is to minimize the effects of bias caused by uneven population densities or by unbalanced observation reports near anthropogenic centres^63–65^. Once spatial filtering was completed, we added a buffer around each occurrence record in accordance with the known home range of the species (Supplementary Table S2). This buffer represented the tentative range in which a single individual is likely to move in, ultimately representing the range of each species added together. This range map was subsequently used to determine which species occupied the sampling sites used in the study.

### Environmental modelling

We constructed a species distribution model via the MaxEnt software package (v.3.3.3, Phillips *et al*.^66^). In order to represent pseudo-absences, we chose 10,000 additional data points by random, a number acceptable for similar sample sizes^64,67^, which were outside of the common myna range which were treated as pseudo-absences for common mynas and either presence or absence for the interactor species, depending on whether or not they were located within the range of an interactor. We extracted the individual values of every predictor variable for each presence/absence site from the original variable layer. To reduce model complexity, we used a stepwise backward elimination. We first ran the full model with all 13 predictor variables in MaxEnt. We used clumping to build the models with cross-validation as the estimation method of error rate. The results included percent contribution calculated for each of the predictor variables, allowing us to select the factors with the highest contribution to the model that were not highly correlated (Pearson’s |r| < 0.75) and had a contribution of >1%. Once the final selection of the predictors was complete, we optimized model parameters (i.e., regularization multiplier and combination of feature classes) by running the full model with a series of regularization multipliers (0.25, 0.50, 1, 1.50, 2, 4, 6) and feature class combinations (L, LQ, H, LQH, LQHP, LQHPT; where L = linear, Q = quadratic, H = hinge, P = product and T = threshold) via the R package ‘ENMeval’^68^ which indicates the optimal regularization multiplier and feature class combination in order to avoid overfitting of the model. The final model included five a-biotic variables (bio4, bio11, bio17, IMP and HD) with LQH feature class combination and 1.5 regularization multiplier. Once all model features were selected, we ran the MaxEnt model with 100 replicates.

### Modelling environmental variables with species co-occurrences

After selecting the optimal environmental predictor variables, we aimed to test the effect of including species co-occurrences in the model using the 12 selected local species. However, because common mynas invasions have been shown to be correlated with local species declines^69^, we used local species presence records only from the years 2016–2018 that reflect present time, and exclude historic records that may have been affected by the introduction of common mynas into Israel. We tested for correlation between species records using Pearson’s |r| (<0.75), but none were highly correlated. Therefore, we employed a joint species distribution model (JSDM) following Pollock *et al*.^22^, and fit it with the Markov Chain Monte Carlo Bayesian modelling software JAGS version 4.3.0 run in the R package R2jags version 0.5-7 with R version 3.4.0^70,71^. We ran the models with five chains for 1,000,000 iterations and a burn-in of 15,000 and thinned the samples by a factor of 1,000. Because JSDM runs in the resolution we employed are time-consuming and limited by computing power, we employed 5 cross-validation tests with 60% of the data for model training and 40% used to validate model output. These tests confirmed our findings (mean AUC = 0.897, values between 0.884–0.904; mean sensitivity = 80.53, values between 74.92–84.69; cell size = 0.03).

### Model evaluation

We evaluated model performance with two indices, the threshold-independent average test value of the Area Under the Curve (AUC) of the Receiver Operating Characteristic plot (ROC) and the threshold-dependent model sensitivity (true positives^72^) for the Maximum Sensitivity plus Specificity threshold^73^. AUC values, which measures the overall discrimination ability of the model, range between 0 (lowest predictability) and 1.0 (highest predictability), with 0.5 being predictability made in random. This value is automatically calculated in the MaxEnt algorithm, and we calculated it for the JSDM with the R package ‘pROC’^74^. Additionally, we calculated model sensitivity by counting the number of presence records that were correctly located within the predicted range (no. of true positives/total no. of occurrence *100). The Maximum Sensitivity plus Specificity threshold was generated automatically by MaxEnt and calculated via the ‘pROC’ library for the JSDM. Model sensitivity was calculated for different spatial resolutions (cell size: 0.004, 0.01, 0.02, 0.03 and 0.05) in order for the models to be compared.

In addition to detecting variable contribution, we generated continuous prediction maps for both the MAXENT and JSDM models based on the probability of occurrence. Whereas this option is included within the MaxEnt software package, it was not introduced to the code by Pollock *et al*.^22^. Therefore, we generated a grid throughout the study extent (cell size: 0.004) and extracted the values for each predictor variable per cell. Subsequently, we calculated the probability of occurrence (Mu) of common mynas per each cell by

$$Mu < -\,pnorm(intercept+Bet{a}_{P1}\ast P1+Bet{a}_{P2}\ast P2+\ldots +BetaPn\ast Pn)$$

whereas *BetaPn* is the coefficient for predictor *n*, *Pn* is the predictor value, and normalization is carried out by the ‘pnorm’ function in R^71^. A detailed illustration of the modelling process of both the SDM and JSDM as we employed them is provided in Supplementary Fig. S2.

## Supplementary information

Supplementary information

## Data Availability

Data is available upon request.
