# Supplementary material for: The effect of local species composition on the distribution of an avian invader
Source: Sci Rep. 2019 Nov 1;9:15861. doi: 10.1038/s41598-019-52256-9 (PMC6825180; doi:10.1038/s41598-019-52256-9)
Supplement: Supplementary file 1 — Supplementary information [file 41598_2019_52256_MOESM1_ESM.pdf]

Supplementary material for:

The effect of local species composition on the distribution of an avian invader

Tali Magory Cohen*[1] & Roi Dor*[1]

[1] *School of Zoology, Faculty of Life Sciences, Tel Aviv University, Tel Aviv 69978, Israel*

***Correspondence:** roidor@tauex.tau.ac.il, talimagory@gmail.com

Appendix 1

Tables S1, S2, S3

Figures S1, S2

Tables

Table S1. Details of the resources used to collect species occurrence records used in this study.

**Type**	**Source**
Online database	Global Biodiversity Information Facility (GBIF) (GBIF.org 2015), VertNet (vertnet.org. 2015), SABAP2 [3]
Previous studies	Holzapfel et al. 2006
Governmental agencies^	Israel Nature and Park Authority (INPA)
Citizen Science project*	Birders, schools, amateur birders
Non-Government Organizations	The Israeli Center for Yardbirds, HaMaarag – Israel's National Ecosystem Assessment Program, Society for the Protection of Nature in Israel
Museums	The Steinhardt Museum of Natural History, Israel
Personal observations	Research team
Personal correspondence	C. Holzapfel

^ © Data – Department of Information Systems, INPA
* In collaboration with The Israeli Center for Yardbirds

Table S2. Home ranges of the species used in the study. References include the source for this value; when no exact information for the species was available, a close relative was used, indicated prior to the reference. N – number of observations.

**Common name**		**Abbreviation**	**N before spatial filtering**	**N after spatial filtering**	**Home range [km]**	**Reference**
Common myna	*Acridotheres tristis*	MYN	7,896	762	3	[5]
Hooded crow	*Corvus cornix*	HCR	21,181	7,181	6.4	American crow; [6]
Western jackdaw	*Corvus monedula*	JAC	4,005	1,368	6.4	American crow; [6]
House sparrow	*Passer domesticus*	HSP	17,831	6,613	2	[7]
Rose-ringed parakeet	*Psittacula krameri*	ROS	11,935	4,734	1.2	[8]
Vinous-breasted starling	*Acridotheres burmannicus*	VST	236	133	3	Common myna; [5]
Monk parakeet	*Myiopsitta monachus*	MON	1,304	645	1.2	Rose ringed parrot; [8]
Syrian woodpecker	*Dendrocopos syriacus*	WOO	7,406	1,654	0.089	[9]
Eurasian hoopoe	*Upupa epops*	HOO	8,521	2,860	0.7	[10]
Great tit	*Parus major*	TIT	10,252	2,739	0.134	[11]
Tristram's starling	*Onychognathus tristramii*	TRI	3,013	866	14.25	[12]
Domestic pigeon	*Columba livia*	PIG	10,401	4,330	0.09	[13]
Laughing dove	*Streptopelia senegalensis*	LAU	15,137	5,261	0.09	Domestic pigeon; [13]

Table S3. Mean regression coefficients of the probit model for each environmental predictor for each species.

Species	Intercept	BIO4	BIO11	BIO17	HD	IMP
Common myna	-1.80	-0.28	0.31	0.13	0.29	0.36
Syrian woodpecker	-3.34	-0.32	-0.05	0.22	0.07	0.02
Hooded crow	0.13	-0.39	0.15	0.37	0.00	0.16
Great tit	-3.12	-0.37	-0.02	0.18	0.04	0.06
Tristram's starling	-0.11	0.98	-0.03	-0.07	0.15	-0.01
Vinous-breasted starling	-3.90	-0.45	0.36	0.14	0.22	0.15
Rose-ringed parakeet	-2.02	-0.30	0.32	0.29	0.16	0.23
Monk parakeet	-3.08	-0.27	0.26	0.06	0.15	0.21
House sparrow	-1.01	0.04	0.23	0.23	0.11	0.33
Western jackdaw	-0.52	-0.20	0.12	0.25	0.56	0.02
Eurasian hoopoe	-1.72	-0.08	0.20	0.09	0.07	0.16
Laughing dove	-2.85	0.14	0.17	0.26	0.04	0.13
Domestic pigeon	-2.92	0.01	0.14	0.14	0.02	0.16

Figures

Figure S1. Response curves created by a MaxEnt model. The curves show the mean response of the 100 replicate MaxEnt runs (marked in red) surrounded by mean +/- one standard deviation marked in blue. These responses are created individually by different models using only the corresponding variable and show its effect on the logistic prediction made by the model. Predictors abbreviations are detailed in Table S2.

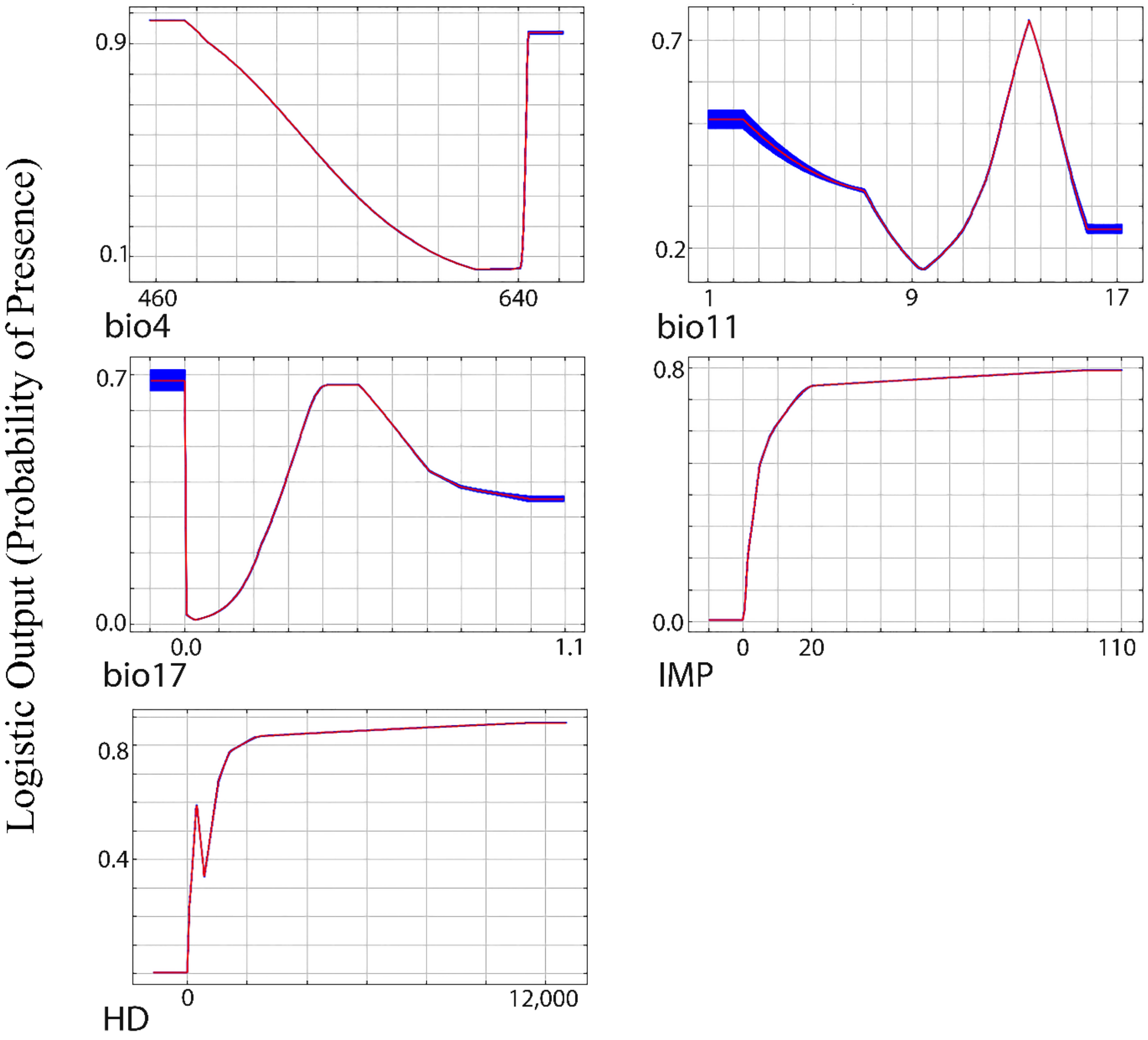
Logistic Output (Probability of Presence)
0.9
0.1
460
640
bio4
0.7
0.2
1
9
17
bio11
0.7
0.0
0.0
1.1
bio17
0.8
0.0
0
20
110
IMP
0.8
0.4
0
12,000
HD

Figure S2. A detailed illustration of the modelling process of both the SDM and JSDM as employed in this study.

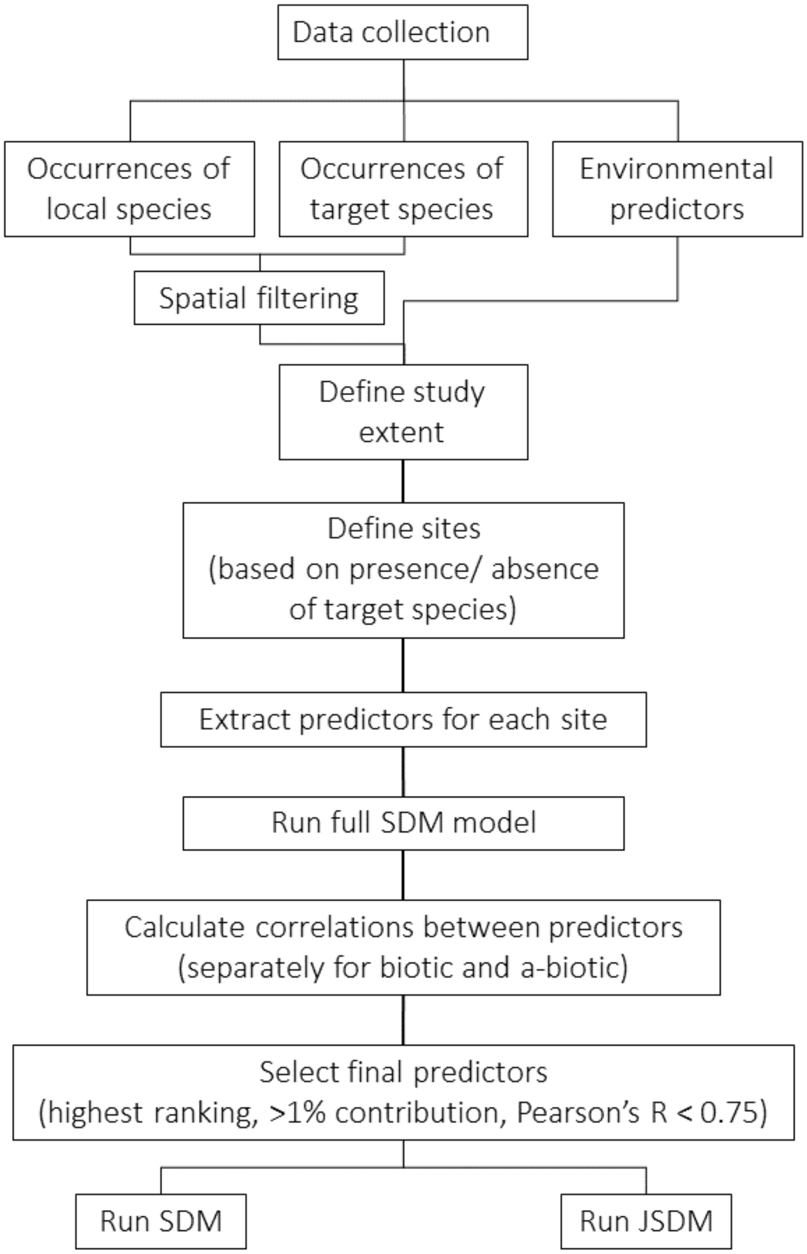

References

1. GBIF.org. *GBIF Occurrence Download* (2015).

2. vertnet.org. *Acridotheres tristis* (2015). Available at: http://portal.vertnet.org/search?q=acridotheres+tristis. (Accessed: 21st October 2015)

3. Brooks, M. Southern African Bird Atlas Project 2: Full protocol records. Version 1.14. (2017).

4. Holzapfel, C., Levin, N., Hatzofe, O. & Kark, S. Colonisation of the Middle East by the invasive Common Myna Acridotheres tristis L., with special reference to Israel. *Sandgrouse* **28**, 44 (2006).

5. Peneaux, C. & Griffin, A. S. Opportunistic observations of travel distances in common mynas (Acridotheres tristis). *Canberra Bird Notes* **40**, 228–234 (2016).

6. Yaremych, S. A., Novak, R. J., Raim, A. J., Mankin, P. C. & Warner, R. E. Home range and habitat use by American Crows in relation to transmission of West Nile virus. *Wilson Bull.* 232–239 (2004).

7. Summers-Smith, D., Christie, D. . & Garcia, E. F. . House Sparrow (Passer domesticus). in *Handbook of the Birds of the World Alive* (eds. del Hoyo, J., Elliott, A., Sargatal, J., Christie, D. . & de Juana, E.) (Lynx Edicions, 2018).

8. Strubbe, D. & Matthysen, E. A radiotelemetry study of habitat use by the exotic Ring-necked Parakeet Psittacula krameri in Belgium. *Ibis (Lond. 1859).* **153**, 180–184 (2011).

9. Pasinelli, G. Oaks (Quercus sp.) and only oaks? Relations between habitat structure and home range size of the middle spotted woodpecker (Dendrocopos medius). *Biol. Conserv.* **93**, 227–235 (2000).

10. Krištín, A. & Kirwan, G. . Common Hoopoe (Upupa epops). in *Handbook of the Birds of the World Alive* (eds. del Hoyo, J., Elliott, A., Sargatal, J., Christie, D. . & de Juana, E.) (Lynx Edicions, 2018).

11. Gosler, A., Clement, P. & Christie, D. . Great Tit (Parus major). in *andbook of the Birds of the World Alive* (eds. del Hoyo, J., Elliott, A., Sargatal, J., Christie, D. . & de Juana, E.) (Lynx Edicions, 2018).

12. Spiegel, O. & Nathan, R. Incorporating dispersal distance into the disperser effectiveness framework: frugivorous birds provide complementary dispersal to plants in a patchy environment. *Ecol. Lett.* **10**, 718–728 (2007).

13. Baptista, L. ., Trail, P. ., Orblit, H. . & Boesman, P. Rock Dove (Columba livia). in *Handbook of the Birds of the World Alive* (eds. del Hoyo, J., Elliott, A., Sargatal, J., Christie, D. . & de Juana, E.) (Lynx Edicions, 2018).
